# Prevalence and Predictors of Guideline Concordant Pediatric Obesity Care: A Narrative Review

**DOI:** 10.1007/s13679-026-00712-9

**Published:** 2026-04-14

**Authors:** Colin J Orr, Aspen E Streetman, Ivan A Carruyo, Amy L Beck, Katie Queen, Tierra Sanders, William J Heerman

**Affiliations:** 1 Department of Pediatrics, Vanderbilt University Medical Center, 2146 Belcourt Ave, Office Suite 212, Nashville, TN 37212 USA; 2 Department of Pediatrics, University of California San Francisco, San Francisco, CA USA; 3 Department of Pediatrics, FMOL Health - Our Lady of the Lake Children’s Health, Louisiana State University Health Sciences, Pennington Biomedical Research Center, Baton Rouge, LA USA

**Keywords:** Food insecurity, Parental feeding behaviors, Pediatric obesity, Child health

## Abstract

**Purpose of Review:**

To summarize the current state of adoption of the 2023 American Academy of Pediatrics (AAP) Clinical Practice Guideline (CPG) for the evaluation and treatment of childhood obesity, identify barriers and facilitators to guideline-concordant care, and highlight gaps in research and clinical practice.

**Recent Findings:**

The AAP CPG outlines 13 Key Action Statements (KASs) for the screening, diagnosis, and treatment of pediatric obesity. Evidence suggests variable uptake across KASs. For example, BMI measurement (KAS 1) is limited by missed well-child visits and insurance disparities, while comprehensive evaluation (KAS 2) faces challenges in mental health and social needs screening. Screening for comorbidities such as dyslipidemia, diabetes, and metabolic dysfunction-associated steatoic liver disease (MASLD) remains low despite longstanding recommendations. Treatment-related KASs (11–13) show significant gaps: referrals to intensive health behavior and lifestyle interventions are infrequent, pharmacotherapy use is < 2% among eligible adolescents, and bariatric surgery referrals remain rare. Barriers include provider hesitancy, resource limitations, and systemic inequities; facilitators include electronic health record integration, multidisciplinary teams, and expanded insurance coverage.

**Summary:**

Despite strong evidence supporting early and intensive treatment of pediatric obesity, guideline adoption remains inconsistent. Addressing structural barriers, improving provider education, and leveraging health system innovations are critical for implementation. Future research should evaluate effective implementation strategies, long-term outcomes of pharmacotherapy, and approaches to adapt guidelines to local contexts.

## Introduction

Over the last three decades, childhood obesity has become the most common chronic disease of childhood. In the United States, approximately 20% of children and adolescents have obesity, and 33% have overweight [1]. In some population sub-groups, the prevalence of obesity is approaching 30%, and the prevalence of overweight is approaching 50% [2, 3]. Childhood obesity leads to both contemporaneous and long-term health consequences, including type 2 diabetes, metabolic dysfunction-associated fatty liver disease, cardiovascular disease, and even some forms of cancer [4, 5]. In addition, childhood obesity is associated with lower quality of life and mental health concerns, especially among adolescents [6–8]. The persistently high rates of childhood obesity and its myriad complications have created a public health crisis. Yet, we are not without evidence-based treatment and prevention strategies. Successfully treating obesity during childhood can reduce the risk of developing type 2 diabetes by 80%, hypertension by 60%, and dyslipidemia by 80% [9].

In 2023, the American Academy of Pediatrics (AAP) published a comprehensive Clinical Practice Guideline (2023 CPG) for the evaluation and treatment of obesity in children and adolescents [10]. The 2023 CPG outlines 13 key action statements (KASs) for the screening, diagnosis, and treatment of children with overweight or obesity (Table 1). The 2023 CPG is based on the best scientific evidence for the evaluation and management of children with overweight or obesity [11, 12]. Like all medical practice guidelines, some recommendations are likely to have been widely adopted by pediatric healthcare providers already, while others may be less commonly adopted. The literature suggests that most guidelines average seventeen years for research evidence to be integrated into clinical practice [13]. Previous literature suggests a number of factors that drive uptake of medical practice guidelines, including provider, patient, community, societal, and health care system characteristics [14]. While all guidelines are meant to create aspirational goals for care, the degree to which it is feasible to implement all of the components of the 2023 CPG remains unclear, especially among populations at highest risk for obesity [15–17].

**Table 1:** Summary of 13 key action statements from the AAP 2023 Childhood Obesity Clinical Practice Guideline [10]. Recommendations are organized by focus area and include strength of recommendation and evidence grade as reported in the guideline. IHBLT = intensive health behavior and lifestyle treatment; BMI = body mass index

Key Action Statement	Focus Area	Summary of Recommendation	Strength / Evidence Grade
1	Diagnosis and Measurement	Screen all children ≥ 2 years annually for overweight and obesity using BMI percentile for age and sex.	Moderate / B
2	Evaluation	Evaluate children with overweight or obesity for comorbidities by using a comprehensive history, mental and behavioral health screening, social determinants of health evaluation, and diagnostic studies.	Strong / B
3	Comorbidities	In children ≥ 10 years old with obesity, obtain lipids, glucose, and liver enzymes. In children ≥ 10 years old with overweight obtain lipids.	Strong / B
4	Comorbidities	Provide treatment for overweight or obesity and comorbidities concurrently.	Strong / A
5	Comorbidities	Obtain fasting lipid panel in children ≥ 10 years old with overweight and obesity. May obtain fasting lipid panel for children 2–9 years old.	Strong / B (≥ 10 years) Moderate/ C (2–9 years)
6	Comorbidities	Evaluate for prediabetes and/or diabetes with fasting plasma glucose, 75-g oral glucose tolerance test, or hemoglobin A1c	Moderate / B
7	Comorbidities	Evaluate for NAFLD by obtaining alanine transaminase (ALT)	Strong / A
8	Comorbidities	Evaluate for hypertension by measuring blood pressure at every visit starting at age 3 in children with overweight or obesity.	Moderate / C
9	Treatment	Obesity should be treated using the principles of the medical home that uses a family-centered and non-stigmatizing approach. Treatment should address biological, social, and structural drivers.	Strong / B
10	Treatment	Use motivational interviewing when treating overweight or obesity.	Moderate / B
11	Treatment	Children with overweight and obesity should be referred to intensive health behavior and lifestyle treatment (IHBLT). This is most effective when > 26 h of face-to-face, family-based, and multicomponent treatment.	Moderate /B (≥ 6 years) Moderate / C (2–5 years)
12	Treatment	Children ≥ 12 years old with obesity should be offered weight loss pharmacotherapy, considering risks and benefits, and as an adjunct to IHLBT.	B
13	Treatment	Children ≥ 13 years old with severe obesity should be referred for evaluation for metabolic and bariatric surgery.	C

Describing the current state of the 2023 childhood obesity CPG’s adoption and the specific facilitators and barriers to adoption of each of its key action statements is a foundational part of understanding the 2023 CPG’s impact on childhood obesity prevention and treatment. Thus, we undertook a narrative review of the scientific literature to summarize the available data on 2023 CPG adoption, with the goals of characterizing the current state of childhood obesity care in the United States and identifying practice and research gaps.

### Diagnosis and Measurement

### Key Action Statement 1: Annual BMI Screening

KAS1 recommends that pediatric healthcare providers measure height and weight, calculate BMI, and assess BMI percentile using standardized growth charts, at least annually for children 2–18 years old [10]. No recent data quantify how often pediatric healthcare providers measure height and weight during well-child visits. Large epidemiologic studies using electronic health record (EHR) data suggest these measurements are routinely collected, with only a small percentage of implausible values [18–20]. However, it is unclear how often providers actively reference BMI during the visit. Several studies highlight gaps between recorded BMI values and corresponding diagnostic codes [21, 22]. For example, a quality improvement project increased pediatric healthcare providers’ documented diagnosis of obesity from 50% at baseline to 71% at follow-up.^21^ Similar improvements have been reported with the use of clinical decision support tools across various healthcare settings [22].

The 2023 CPG emphasizes that care for children with overweight or obesity is best delivered within a primary medical home model, that ensures continuity and comprehensive management of chronic conditions such as obesity (KAS 9). Despite these recommendations, attendance at well-child visits remains inconsistent, creating gaps in obesity screening. For example, a study that used data from the OCHIN research network and Virginia Commonwealth University Health system, representing community health centers, federally qualified health centers, rural hospitals, and Critical Access Hospitals in 19 states, found that in the first six years of life, the most frequently missed well child visits were at 15 months (56%), 18 months (41%), and 4 years (19%).^23^ In that same study, insurance status was a significant predictor of attendance: children with public insurance had 38% lower odds of completing well-child visits compared to those with private insurance [23]. Data from the Medicaid Expenditure Panel revealed that the COVID-19 pandemic reduced overall well-child visit rates from 66.6% in 2018–2019 to 58.6% in 2020 [24]. Although rates stabilized in 2021, disparities persisted as attendance among Black non-Hispanic and Hispanic children was approximately 25% lower than among White children.

### Summary and Strategies to Improve Diagnosis and Management

In summary, systematic data on BMI measurement frequency are lacking while missed well-child visits remain the primary barrier to routine BMI screening. One potential system-level improvement would be supporting clinicians in recognizing and documenting BMI values consistent with overweight and obesity.

## Evaluation

### Key Action Statement 2: Screening for Mental Health and Health Related Social Needs

In addition to conducting routine aspects of pediatric well-care, like a comprehensive history and physical examination, KAS2 highlights the importance of screening for and addressing both mental and behavioral health issues and the social determinants of health when treating childhood obesity [10].

### Mental Health Screening

In recent years, there has been increasing awareness of the need to improve screening rates for depressive and anxiety symptoms in pediatric primary care settings [25, 26]. Prior to 2020, rates of mental health screening were extremely low [27]. For example, a recently published cross-sectional and population-based analysis using 2008–2018 data from the U.S. Centers for Disease Control and Prevention’s (CDC) National Ambulatory Medical Care Survey found that the overall prevalence of screening for depression in adolescents was less than 4% [28]. In that same analysis, children with obesity had the lowest reported frequency of depression screening at 2.89%. In addition, the AAP’s Periodic Survey in 2013 showed that the percentage of all pediatricians surveyed who screen for depression was below 25% nationwide [29]. More recent data about screening for mental health conditions among children with obesity have not been published, and an important opportunity for future research would be to evaluate how variation in practice type and location (e.g., academic vs. community practice, rural vs. urban location) may be associated with differential screening rates.

### Health-Related Social Needs

Increasingly, pediatric healthcare practices are integrating more structured health-related social needs screening into routine clinical care [30]. The recognition that these needs are associated with the incidence of childhood obesity and the heterogeneity of treatment effect of successful interventions has been well-described [31 ].The routine incorporation of social needs screening was first widely adopted for food insecurity screening. This was driven by the AAP’s 2015 recommendation that pediatric healthcare providers should universally screen patients for food insecurity [32]. Recently, social needs screeners have included other domains like financial hardship, housing instability, and transportation security (among others) [33].

Despite the increasing recognition of the importance of health-related social needs, especially in the context of obesity treatment and prevention, overall screening rates remain highly variable. One recent scoping review synthesized 42 implementation studies (29 in primary care; 17 in pediatric primary care) and reported wide practice-level variation in health-related social needs screening, with rates ranging from ~ 5% to ~ 93% across individual clinics [34]. In addition, data from the AAP Periodic Survey ion 2019 indicate that 76.7% of pediatric healthcare providers screen for health-related social needs, but only 12.6% use a validated screening tool [35]. The variability in screening practices is driven by several factors, including patient hesitancy to answer sensitive screening items and provider hesitancy to screen for social challenges for which they cannot provide meaningful intervention [36–38]. For example, patient non-response to screening items is common (nearly 10%) and more common among certain population sub-groups [39].

### Summary and Strategies to Improve Screening Rates

Several successful projects, especially in academic medical centers, have led to improvement of screening for mental health and health-related social needs [40, 41]. The integration of screening tools for health-related social needs into electronic health records has been shown to substantially increase screening rates [40]. In addition, focused quality improvement initiatives have also led to increased screening rates [41].

In summary, the 2023 CPG emphasizes screening for mental health concerns and health-related social needs as part of comprehensive obesity care. Screening rates for depression, anxiety, and social needs remain variable, with limited use of validated tools. Integration into electronic health records and targeted quality improvement initiatives show promise, but substantial gaps persist, highlighting opportunities for system-level interventions.

### Key Action Statements 3 Through 9: Screening for Comorbidities

The 2023 CPG recommends screening for lipid abnormalities with a fasting lipid panel, screening for abnormal glucose metabolism with a fasting glucose, oral glucose tolerance test or hemoglobin A1C (HgA1C) and screening for metabolic dysfunction-associated steatotic liver disease (MASLD) with an alanine transaminase (ALT) test. The 2023 CPG also recommends evaluating all children ages 3 and older with overweight or obesity for hypertension by assessing blood pressure at every visit [10].

### Overall Screening Rates

Available evidence, although limited, indicates that most children with obesity in the United States are not screened in accordance with the new 2023 CPG recommendations [42, 43]. One study among children with obesity that used EHR data from 2020 to 2022 found that only 13% of children were screened for dyslipidemia, pre-diabetes/type 2 diabetes, and liver dysfunction, and 74% of children were not screened at all [42]. In another study from 20,172,020, children with obesity who identified as Black or Hispanic had increased odds of being screened for dyslipidemia, diabetes, liver dysfunction, completing biochemical screening, and being screened for all conditions compared to children who identified as White [43].

### Lipid Screening

Research examining predictors of lipid screening is comparatively more robust, likely because universal lipid screening for children aged 9–11 years, regardless of BMI category, has been recommended by the AAP since 2011 [44]. However, adherence remains low. For example, a recent retrospective analysis of 8.4 million children using the MarketScan Commercial and Medicaid claims databases, revealed only 17.5% of healthy children underwent lipid screening, and high-risk children were nearly twice as likely to be screened [45]. In another study conducted in Utah, among approximately 64,000 children aged 9–11 years, only 3.5% were screened for lipid abnormalities between 2009 and 2011 and 2013–2015, with no significant improvement over time, although children with overweight or obesity were more likely to be screened [46]. Similarly, among approximately 270,000 Medicaid-enrolled children in Florida who turned 12 between 2015 and 2019, only 12.3% of those with obesity and 7.7% of those without obesity were screened before 12 [47]. In a study published in 2025, 18% of children were screened for dyslipidemia [42]. The prevalence of completed screening was higher among older children, females, and children with a more severe obesity category.

### Screening for Prediabetes/Type 2 dDiabetes

Screening rates for prediabetes and type 2 diabetes are similarly low. In a study of over one million children aged 10–19 years receiving care at 800 ambulatory sites in the United States between 2019 and 2021, only 13% of those with obesity were screened for prediabetes or type 2 diabetes [48]. A smaller study of approximately 34,000 youth aged 10–18 years with overweight or obesity in Pennsylvania found 21% had diabetes screening ordered while14% completed screening, and that onsite phlebotomy increased the likelihood of screening completion [49]. A recent study found 22% of children were screened for prediabetes/type 2 diabetes [42]. Similar screening patterns as for dyslipidemia were observed with older children, females, children with a more severe obesity category being more likely to be screened for prediabetes/type 2 diabetes [42].

### Screening for MASLD

Screening rates for MASLD are also low. In a large national EHR study from 2019 to 2021, among ~ 920,000 U.S. children aged 9–19 with excess weight, only 13% had ≥ 1 ALT result—and among those with obesity, ~ 14% were screened, underscoring low uptake of recommended MASLD screening [50]. Recent data from 2025 also aligned with this finding, where 19% of children with obesity were screened with an ALT [42].

### Screening for Hypertension

Appropriate hypertension screening is also low in pediatric primary care, while the prevalence of hypertension is higher than historically realized [51–54]. In 2017, the AAP published a clinical practice guideline for high blood pressure among children [55]. In 2022, a study was conducted among 59 pediatric primary care practices. This study found that approximately 2% of patients had all steps in measuring blood pressure completed correctly (i.e., undergoing 3-limb and ambulatory blood pressure monitoring) [56]. This study also found that 10% of patients who presented to clinic with three elevated blood pressure readings had a diagnosis of hypertension documented in the electronic health record [56].

### Summary and Strategies to Improve Screening Rates

Despite strong recommendations in the 2023 CPG, adherence to cardiometabolic screening for children with overweight and obesity remains low. Across multiple large-scale studies, fewer than one in five children receive recommended screening for dyslipidemia, prediabetes/type 2 diabetes, or MASLD, and most are not screened at all. Collectively, these findings underscore an urgent need for system-level interventions (ex. on site phlebotomy services), workflow integration, and policy strategies to improve adherence to guideline-based screening and reduce missed opportunities for early detection and management of cardiometabolic risk in pediatric populations.

## Key Action Statements 10–13: Treatment

The 2023 CPG identifies a range of treatment approaches, based on strong evidence, including office-based motivational interviewing (MI) and family-based care, intensive health behavior and lifestyle treatment (IHBLT), anti-obesity medications, and bariatric surgery [10].

### Motivational Interviewing

MI is the first treatment strategy discussed in the 2023 CPG. MI is an adaptable, patient-centered communication style that identifies and supports patients and families [57]. There are no available studies about the prevalence of routine use in pediatric primary care settings.

### Referral to IHBLT

The 2023 CPG recommends strongly against watchful waiting in the treatment of childhood overweight and obesity. Rather it recommends prompt referral to IHBLT or the highest level of care available. The 2023 CPG states that IHBLT is most successful when ≥ 26 h of face-to-face contact are provided over 3 to 12 months, using a family-centered treatment paradigm. The 2023 CPG also acknowledges that this level of intensive intervention may not be feasible in many settings, in which case other interventions (e.g., referral to a nutritionist) would be appropriate [10].

Recent data about the availability of IHLBT that meet the 26-hour standard are not available [58]. The majority of studies focus evaluate referrals to any type of weight-management program. For example, in an analysis of data from 2017, only 15% of clinicians reported referring more than 75% of their patients with obesity to a weight‑management program [59]. Female clinicians were more likely to refer, whereas older clinicians were less likely. Referral patterns also reflected patient household resources: clinicians serving lower‑resourced communities had reduced odds of referring to subspecialists, while those serving middle‑resourced families had higher odds of referring patients to weight‑management services [59].

A study that used data from 2021 to 2024 to examine changes in treatment for pediatric obesity before and after the publication of the 2023 CPG found that 9.7% of patients received a referral for nutrition services [60]. The study also found that children identifying as Black, Hispanic, or Asian had higher odds of referral to a nutritionist. No statistically significant change in referral patterns was observed after the publication of the guideline (OR 1.05 [0.98–1.12]). Variations in referral patterns were also observed by provider training with family medicine, internal medicine, and nurse practitioner/physician assistants having decreased odds of referral to nutrition services compared to pediatricians. Residents had higher 39% higher odds of making a referral compared to attending pediatricians [60].

Patient characteristics have been shown to predict participation in weight management therapies. A retrospective chart review of patients referred to a pediatric weight loss clinic found that older children age (OR 0.93 [0.91-096]), male sex (0.74 [0.6–0.91]), and those with public insurance (OR 0.77 [0.6–0.98]) had decreased odds of attending the clinic’s orientation while families identifying as Hispanic and living in the city limits had 70% and 39% higher odds of attending the clinic’s orientation (first visit) [61]. An obesity treatment study from Massachusetts demonstrated how health-related social needs can negatively impact treatment for obesity [62]. The study found that specific health-related social needs (such as housing instability) and the number of social needs were negatively associated with the number of contact hours with a pediatric obesity treatment intervention. In the study, participants who experienced housing instability or had three or more unmet social needs had, on average three fewer contact hours with the intervention than participants without housing instability [62].

### Utilization of Pharmacotherapy for Pediatric Obesity

Pharmacological management of pediatric obesity has been revolutionized by glucagon-like peptide-1 receptor agonists (GLP-1RAs), namely semaglutide which was approved for the treatment of obesity in adolescents aged 12–17 in December 2022. GLP-1RAs have been proven to be extremely effective in the treatment of pediatric obesity in randomized clinical trials [63].

Dispensing of GLP-1RAs to adolescents and young adults increased substantially between 2020 and 2023, yet coverage gaps persist, particularly for obesity indications [64]. For example, in one study of over 300,000 children and adolescents with obesity, less than 0.4% of patients were prescribed pharmacotherapy to treat their obesity [60]. However, investigators also found 65% higher odds of medication prescription after the publication of the 2023 CPG. In another study, female patients and patients with higher BMI were more likely to be prescribed pharmacotherapy to treat obesity; however, patient’s identifying as Black or Native American were less likely to be treated with pharmacotherapy for their obesity [65].

Access to GLP-1RAs for adolescents remains constrained by affordability and insurance coverage limitations, despite growing clinical adoption. National estimates suggest that approximately 17 million youth aged 12–25 meet eligibility criteria for GLP-1RA therapy, but lack of insurance and inconsistent coverage remain major barriers to access [66]. Even within specialized pediatric weight management programs, cost and coverage variability are cited as key obstacles to equitable prescribing, despite efforts to standardize clinical decision-making [67]. Collectively, these findings underscore the need for policy reforms and payer engagement to ensure equitable access to evidence-based pharmacotherapy for pediatric obesity.

### Metabolic and Bariatric Surgery

Surgical interventions for severe obesity are effective [68]; however, multiple barriers limit their use. In a single‑site study (2017–2019), of ~ 1,200 children who met referral criteria for metabolic and bariatric surgery (MBS), only 12% were referred, and just 22% of those referred ultimately underwent surgery [69]. Eligibility was similar for boys and girls, but female patients had higher odds of referral. Black and Hispanic children were more likely to meet eligibility criteria, yet referral rates did not differ statistically by race/ethnicity. By insurance status, children with public insurance were more likely to meet criteria, while referral rates did not differ by coverage [69].

A study of national trends (HCUP/Kids’ Inpatient Database, 2009–2017) reported non‑significant increases in the proportion of MBS procedures among Black (from 12.1% to15.8%) and Hispanic (from 16.8% to 22.1%) children [70]. Another analysis found ~ 50% lower odds of undergoing MBS among Black or Hispanic patients compared with White patients [71]. More recently (2017–2023), MBS increased annually by ~ 2.26% among Black children and by ~ 1% among Hispanic children [72]. Across these studies, girls are disproportionately represented among pediatric MBS recipients, suggesting a sex‑related disparity that warrants further investigation [73]. Geographic variation is also evident, with more procedures performed at teaching hospitals and in the Northeast and South Census regions [74].

### Summary and Strategies to Improve Treatment for Pediatric Obesity

In summary, the 2023 CPG recommends a comprehensive approach to obesity treatment, including motivational interviewing, IHBLT, pharmacotherapy, and bariatric surgery. Despite strong recommendations, referrals to IHBLT and use of anti-obesity medications remain low, with significant variation by patient and provider characteristics. Access to GLP1-RAs and bariatric surgery is further limited by cost, insurance coverage, and systemic barriers, underscoring the need for policy and implementation strategies to improve access to these evidence-based treatment approaches.

## Discussion

Despite strong evidence supporting early and intensive treatment of pediatric obesity, adoption of the AAP’s 2023 CPG for Pediatric Obesity remains inconsistent. However, because the 2023 CPG was only recently published, numerous aspects of its implementation have not yet been assessed. Future work should focus on (1) developing surveillance systems to measure guideline adoption, with particular focus on facilitators and barriers; (2) using implementation science approaches to support wide-spread uptake of the guidelines; and (3) conducting evaluations of various aspects of the guidelines to support future refinement in subsequent iterations. Informing pragmatic and evidence-based childhood obesity prevention and treatment should remain a top priority for both public health and research.

There are a variety of factors that contribute to limited uptake of clinical practice guidelines. Using a theoretical framework published by Cabana et al. [14], we can identify a variety of clinician barriers to uptake of the 2023 CPG that arise from knowledge, attitudes, and external constraints. First, knowledge barriers include variable awareness and familiarity with key action statements (e.g., annual screening; referral to IHBLT with ≥ 26 contact hours), which can be uneven across primary care settings and among non-pediatric specialists who see children. Similar to other guidelines, limited dissemination and the length/complexity of the 2023 CPG may be impediments to guideline adherence [75–77]. Second, attitudinal barriers include disagreement with recommendations (particularly around early pharmacotherapy and bariatric referral), low self‑efficacy to deliver family-based behavioral interventions at the recommended intensity, and outcome expectancy when clinicians doubt real-world effectiveness in resource-constrained contexts. In addition, inertia of previous practice further slows change—even after guideline release, absolute treatment rates remain low. Third, external barriers are substantial. These include scarcity of IHBLT programs [58], logistical constraints (transportation, missed school/work), reimbursement challenges, workforce capacity, and administrative burdens such as prior authorization for anti‑obesity pharmacotherapy. For pharmacotherapy specifically (e.g., GLP‑1 receptor agonists for adolescents ≥ 12 y), barriers include payor coverage variability, cost, long‑term safety concerns, and programmatic infrastructure for monitoring—even though prescribing has increased modestly since the 2023 CPG’s release. Finally, pervasive weight stigma within clinical encounters can undermine engagement and adherence, highlighting the need for systematic training in person‑first language and empathetic counseling as part of implementation [78, 79]. Collectively, these barriers—mapped to Cabana’s taxonomy—suggest that improving uptake will require multi‑level strategies that simultaneously address clinician capability and motivation and the structural environment in which care is delivered.

Table 2 summarizes priority research gaps that constrain uptake of the 2023 CPG. Aligned with Cabana et al.’s framework [14], gaps cluster across knowledge (awareness, familiarity), attitudes (agreement, self‑efficacy, outcome expectancy), and external barriers (access, payment, care coordination, and measurement). Addressing these gaps through pragmatic implementation trials, policy evaluations, and multi-level interventions is likely to improve guideline concordance and real‑world effectiveness.

**Table 2:** Key research gaps affecting uptake of the 2023 AAP Childhood Obesity CPG. Domains are organized using Cabana et al.’s taxonomy (knowledge, attitudes, external barriers). IHBLT = intensive health behavior and lifestyle treatment; GLP‑1Ra = glucagon‑like peptide‑1 receptor agonist

Domain	Research gap	Why it matters for uptake	Illustrative evidence need
Knowledge: Awareness & familiarity	How best to simplify/operationalize 2023 CPG content for busy primary care workflows (e.g., concise decision pathways, EHR prompts).	Reduces cognitive load; increases consistent application during brief visits.	Pragmatic trials of EHR-integrated decision support vs. usual dissemination.
Knowledge: Training & competencies	Effective models to build self‑efficacy for delivering IHBLT at ≥ 26 h across diverse settings.	Clinician confidence predicts implementation; IHBLT dose is a cornerstone of the 2023 CPG.	Implementation studies comparing team‑based primary care curricula and community‑partnered delivery.
Attitudes: Agreement & outcome expectancy	Real‑world effectiveness of tiered interventions (lower‑dose behavioral care vs. full IHBLT).	Many practices cannot meet 26 + hours; need evidence on stepped‑care approaches.	Comparative effectiveness across intensity levels, stratified by social risk.
Attitudes: Risk–benefit perceptions	Long‑term safety, adherence, and discontinuation outcomes for adolescent GLP‑1Ra therapy, integrated with behavioral care.	Addresses clinician hesitancy and informs shared decision‑making.	Prospective cohorts with ≥ 2–5‑year follow‑up including adverse events and weight trajectories.
External: Access & infrastructure	Strategies to expand IHBLT availability (financing, workforce, telehealth, community partnerships) in underserved areas.	Supply constraints are a primary bottleneck; tele‑IHBLT may mitigate logistics.	Policy‑evaluation studies of reimbursement reforms; trials of tele‑delivered IHBLT.
External: Payment & policy	Impact of reimbursement policies (e.g., new billing codes) and prior authorization on timely initiation of evidence‑based treatments.	Administrative barriers delay care; payment drives implementation.	Natural experiments before/after code adoption; multi‑state claims analyses.
External: Care coordination	Effective models of care that integrate pediatricians, RDs, behavioral health, and community health workers in primary care.	Team‑based approaches may improve reach and outcomes.	Hybrid effectiveness–implementation trials of coordinated primary care clinics.
External: Equity & stigma	Interventions to reduce weight stigma and improve trust/engagement among families facing social risk.	Stigma reduces care-seeking and adherence; equity is central to 2023 CPG goals.	Cluster trials of stigma‑reduction training; qualitative studies on family experiences.
Monitoring & measurement	Standardized implementation metrics (e.g., guideline concordance, time to treatment, dose delivered) and EHR phenotypes for tracking uptake.	Enables benchmarking and rapid-cycle quality improvement.	Consensus development and validation of pediatric obesity implementation measures.

As clinicians and health systems move toward implementing guideline concordant-concordant care into practice, it will be important to consider the experiences of patients and parents. The Health Belief model is one theoretical framework that can be used to explore the perspectives of patients and parents [80]. Perspectives on the guidelines are likely influenced by perceived risk, severity, susceptibility, barriers, benefits, and self-efficacy. Early evidence suggests that parental barriers to guideline-concordant pediatric obesity include knowledge of the guidelines and varying degrees of guideline acceptance, especially around the use of medications and surgery for the treatment of obesity [81]. Parental perspectives on the guidelines can be nuanced and influenced by a parent’s BMI and their own experiences with obesity treatment. As we work toward implementing the guidelines into clinical practice it will be important to continue to engage parents with diverse lived experiences, as well as patients to gain a more holistic understanding of the barriers and facilitators to guideline concordant care to improve the health of children with obesity.

## Conclusion

In conclusion, despite strong evidence supporting early and intensive treatment of pediatric obesity, guideline adoptionremains low. Addressing structural barriers, improving provider education, and leveraging health system innovations are critical for implementation. Future research should evaluate effective implementation strategies, long-term outcomes of pharmacotherapy, and approaches to adapt guidelines to local contexts. Surveillance systems for guideline-concordant care should be developed to monitor progress in translating research evidence into clinical practice as much of the current evidence was published prior to the 2023 guidelines.

## Key References

- Hampl SE, Hassink SG, Skinner AC, et al. Clinical Practice Guideline for the Evaluation and Treatment of Children and Adolescents With Obesity. *Pediatrics*. Feb 1 2023;151(2)doi:10.1542/peds.2022-060640.

○ American Academy of Pediatrics clinical practice guidelines for the evaluation and management of children and adolescents with obesity.

- Pierce SL, Kraus EM, Porter R, Sharifi M, Kompaniyets L, Goodman AB. Assessment of Guideline-Recommended Laboratory Screening for Obesity-Related Chronic Conditions in US Youth 10–18 Years. *Obesity (Silver Spring)*. 2025/10/01 2025;33(10):1921–1929. doi:10.1002/oby.24365.

○ The majority of children with obesity are not screened for dyslipidemia, diabetes, and liver disease as recommended by the 2023 American Academy of Pediatric clinical practice guideline.

- Rodriguez PJ, Do D, Gratzl S, Goodwin-Cartwright BM, Stucky NL, Wright DR. Shifts in US Pediatric Obesity Treatment After the AAP Guidelines. *Pediatrics Open Science*. 2025;1(3):1–12. doi:10.1542/pedsos.2025-000623.

○ Changes in treatment for pediatric obesity before and after the publication of the 2023 American Academy of Pediatric clinical practice guidelines.

## Data Availability

No datasets were generated or analysed during the current study.
